# Emerging materials and transistors for integrated circuits

**DOI:** 10.1093/nsr/nwae040

**Published:** 2024-01-29

**Authors:** Ming Liu, Lian-Mao Peng

**Affiliations:** Lab of Nanofabrication and Novel Device Integration, Institute of Microelectronics, Chinese Academy of Sciences, China; Key Laboratory for the Physics and Chemistry of Nanodevices and Center for Carbon-based Electronics, School of Electronics, Peking University, China

The device technology for integrated circuits (ICs) has received a great deal of attention in the past decade since the mainstream silicon-based complementary metal-oxide-semiconductor (CMOS) field-effect transistor (FET) for modern ICs had been encountering development bottlenecks due to the combination of power constraints, physics, cost and fabrication processes. The main technical challenge is how to construct higher-performance devices with lower power consumption and a smaller footprint. Thus, the developments of emerging digital device technologies have been focused on introducing novel device structures, new semiconducting materials and new architecture to improve performance/integration density and enhance the function of ICs. These efforts can be classified briefly as more Moore, Moore than Moore and beyond CMOS. The first strategy, i.e. more Moore, employs new structures such as gate-all-around (GAA), nanosheet and complementary-FET (CFET) to suppress short-channel effects (SCEs) and improve the drive ability of ultra-scaled Si CMOS transistors. The second strategy is to build MOSFETs using emerging semiconductors, in particular one-/two-dimensional semiconductors with high mobility, ultrathin bodies and good immunity to SCEs. The low-dimensional semiconductor-based FETs exhibit excellent scaling behavior and compatibility with the Si CMOS process, showing great potential for heterojunction integrated three-dimensional ICs in advance technology nodes. The third strategy is to break through the conventional CMOS device and circuit architecture, and develop novel devices based on new state variables—physical quantities that store and transmit the logic state—for computing, interconnects and memory, such as electron dipole, spin, orbital state and light intensity/helicity.

In the past decade, all above three directions have sparked extensive research interest, but the research hotspots concentrate on revolutionary materials and transistors for enabling fundamentally better computing devices at the physical level. To highlight recent developments and crucial issues for future research in digital ICs, we have organized this Special Topic ‘Emerging Materials and Transistors for Integrated Circuits’.

In this Special Topic, we have collected a total of eight papers including five perspective articles, one review article, one interview and one research highlight. Specifically, the first two perspective articles, one from Professor Xinran Wang [1] and the other from Professor Zhiyong Zhang [2], highlight the development of transistors and ICs based on two-dimensional semiconductors and a quasi one-dimensional semiconducting carbon nanotube. The third perspective article from Professor Yunqi Liu [3] focuses on multifunction-oriented high-mobility polymer semiconductors. The fourth perspective article from Professor Chao Zhao [4] proposes the amorphous oxide semiconductors for monolithic 3D dynamic random-access memory (DRAM). The fifth perspective article from Dr. Kaiming Cai [5] focuses on the spin-based magnetic random-access memory (MRAM) for high-performance computing.

In the review article [6], Professor Huaxiang Yin discusses the extension of Si CMOS technology and reviews new-structure transistors for advanced technology node CMOS ICs. In the highlight [7], Professor Xidong Duan introduces the recent progress on two-dimensional transistors from Dr. Chenguang Qiu, who reported an ultra-scaled ballistic FET built on atomic-layer-thick InSe film with performance outperforming all of the other reported 2D FETs and even Si CMOS FETs. In the interview [8], Dr. Defu Wang shows us deep insights into high-frequency and THz electronics from Dr. Dae-Hyun Kim.

Although this Special Topic cannot cover all aspects of emerging semiconducting materials and devices, it gives representative directions by researchers from both academic and industrial communities. We sincerely hope that it will inspire readers from multidisciplinary fields. We are greatly indebted to all contributing authors for the Special Topic, with special thanks to Dr. Dae-Hyun Kim for the wonderful interview. We are also grateful to the editors of *National Science Review* for enthusiastic support. Finally, our warm appreciation goes to the whole editorial team of the journal for their great work.

## References

[1] Li W , Shen H, Qiu H et al. Natl Sci Rev 2024; 11: nwae001. 10.1093/nsr/nwae001 38312376 PMC10837103

[2] Si J , Zhang P, Zhang Z. Natl Sci Rev 2024; 11: nwad261. 10.1093/nsr/nwad261 38312387 PMC10833444

[3] Zhu M , Guo Y, Liu Y. Natl Sci Rev 2024; 11: nwad253. 10.1093/nsr/nwad253 38312388 PMC10833453

[4] Mao S , Wang G, Zhao C. Natl Sci Rev 2024; 11: nwad290. 10.1093/nsr/nwad290 38312381 PMC10833454

[5] Cai K , Jin T, Lew WS. Natl Sci Rev 2024; 11: nwad272. 10.1093/nsr/nwad272 38312380 PMC10833460

[6] Qingzhu Z , Yongkui Z, Yanna L et al. Natl Sci Rev 2024; 11: nwae008. 10.1093/nsr/nwad286 38390365 PMC10883695

[7] Li J , Duan X. Natl Sci Rev 2024; 11: nwad315. 10.1093/nsr/nwad315 38312382 PMC10833443

[8] Wang D . Natl Sci Rev 2024; 11: nwae013. 10.1093/nsr/nwae013 38327852 PMC10849315

